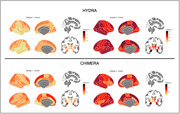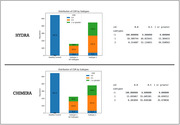# Unraveling Alzheimer's Disease Heterogeneity: A Comparative Analysis Using HYDRA and CHIMERA

**DOI:** 10.1002/alz.093286

**Published:** 2025-01-09

**Authors:** Gordon An, Tammie L.S. Benzinger, Aristeidis Sotiras, Brian A. Gordon

**Affiliations:** ^1^ Washington University in St. Louis, St. Louis, MO USA; ^2^ Mallinckrodt Institute of Radiology, Washington University in St. Louis, St. Louis, MO USA; ^3^ Mallinckrodt Institute of Radiology, Washington University School of Medicine in St Louis, St Louis, MO USA

## Abstract

**Background:**

Alzheimer's Disease can present with heterogenous neurodegenerative patterns. In order to optimize clinical trials and personalized medicine, the identification and characterization of diverse pathological brain patterns associated with AD have become paramount. Optimal approaches to identify such heterogeneity are unknown. The present study employed two distinct clustering approaches, namely HYDRA and CHIMERA, to delineate the spatial pattern of brain atrophy attributable to AD. Methods were applied to MRI scans from the Open Access Series of Imaging Studies (OASIS‐4) project.

**Methods:**

HYDRA uses a convex polytope formed by multiple linear hyperplanes that correspond to various pathological patterns, capturing disease subtypes. CHIMERA assesses the pathological transition by transforming NC distribution to separate transformations matching the disease distribution. While both identify spatial patterns, the distinction lies in HYDRA's discriminative analysis of disease subtypes and CHIMERA's generative nature on disease progression through distribution matching.

**Results:**

Both approaches identified two patterns, or subtypes (Figure 1) with similar CDR results (Figure 2). All subtypes demonstrate marked atrophy within the medial temporal areas, notably the hippocampus. Disparities are evident when assessing subtypes: Subtype‐2 across both methods shows pronounced variations in regions such as superior frontal, middle temporal, parietal cortex, and precuneus, areas paramount in AD pathology. Conversely, HYDRA's Subtype‐1 highlights subtle differences in temporal cortex relative to its Subtype‐2. CHIMERA's Subtype‐1, while mirroring its Subtype‐2 pattern, is less intensified, suggesting an earlier AD stage. Collectively, these patterns concur with recognized AD neuropathological trajectories, pinpointing regions initially impacted in progression. In addition, an in‐depth exploration of subsequent analysis including a longitudinal data evaluation to observe the progression of these patterns over time is slated for later investigation.

**Conclusion:**

Our findings demonstrate data‐driven approaches to derive clinically meaningful patterns of neurodegeneration. A parallel evaluation of both approaches accentuates the robustness of clustering techniques, revealing consistent and overlapping insights into the intricate pathological landscapes of AD. This convergence in findings bolsters confidence in the reliability of such analytical tools in AD research.